# Microbiome predictors of dysbiosis and VRE decolonization in patients with recurrent *C. difficile* infections in a multi-center retrospective study

**DOI:** 10.3934/microbiol.2019.1.1

**Published:** 2019-01-17

**Authors:** Marina Santiago, Lindsay Eysenbach, Jessica Allegretti, Olga Aroniadis, Lawrence J. Brandt, Monika Fischer, Ari Grinspan, Colleen Kelly, Casey Morrow, Martin Rodriguez, Majdi Osman, Zain Kassam, Mark B. Smith, Sonia Timberlake

**Affiliations:** 1 Finch Therapeutics, 200 Inner Belt Rd, Somerville, MA 02143, USA; 2 OpenBiome, 2067 Massachusetts Ave, Cambridge, MA 02140, USA; 3 Division of Gastroenterology, Brigham and Women's Hospital, 75 Francis St, Boston, MA 02115, USA; 4 Department of Medicine (Gastroenterology), Albert Einstein College of Medicine, 1300 Morris Park Ave, Bronx, NY 10461, USA; 5 Division of Gastroenterology, Indiana University School of Medicine, 340 W. 10^th^ St, Indianapolis, IN 46202, USA; 6 Division of Gastroenterology, Icahn School of Medicine at Mount Sinai, 1 Gustave L. Levy Pl, New York, NY 10029, USA; 7 Women's Medicine Collaborative, Brown Alpert Medial School, 222 Richmond St, Providence, RI 02903, USA; 8 Department of Cell, Developmental, and Integrative Biology, University of Alabama at Birmingham, 1720 2^nd^ Ave S, Birmingham, AL 35294, USA; 9 Division of Infectious Diseases, University of Alabama at Birmingham School of Medicine, 1670 University Blvd, Birmingham, AL 35233, USA

**Keywords:** microbiome, fecal microbiota transplant, *Clostridioides difficile*, vancomycin resistant Enterococcus

## Abstract

The gastrointestinal microbiome is intrinsically linked to the spread of antibiotic resistance. Antibiotic treatment puts patients at risk for colonization by opportunistic pathogens like vancomycin resistant Enterococcus and *Clostridioides difficile* by destroying the colonization resistance provided by the commensal microbiota. Once colonized, the host is at a much higher risk for infection by that pathogen. Furthermore, we know that microbiome community differences are associated with disease states, but we do not have a good understanding of how we can use these changes to classify different patient populations. To that end, we have performed a multicenter retrospective analysis on patients who received fecal microbiota transplants to treat recurrent *Clostridioides difficile* infection. We performed 16S rRNA gene sequencing on fecal samples collected as part of this study and used these data to develop a microbiome disruption index. Our microbiome disruption index is a simple index that is predictive across cohorts, indications, and batch effects. We are able to classify pre-fecal transplant vs post-fecal transplant samples in patients with recurrent *C. difficile* infection, and we are able to predict, using previously-published data from a cohort of patients receiving hematopoietic stem cell transplants, which patients would go on to develop bloodstream infections. Finally, we also identified patients in this cohort that were initially colonized with vancomycin resistant Enterococcus and that 92% (11/12) were decolonized after the transplant, but the microbiome disruption index was unable to predict such decolonization. We, however, were able to compare the relative abundance of different taxa between the two groups, and we found that increased abundance of Enterobacteriaceae predicts whether patients were colonized with vancomycin resistant Enterococcus. This work is an early step towards a better understanding of how microbiome predictors can be used to help improve patient care and patient outcomes.

## 1. Introduction

Antibiotic-resistant bacterial infections are a major public health threat with over 2 million Americans infected each year [1]. Most antibiotic resistant infections are transmitted in the community, but most antibiotic use occurs in hospitals, where the widespread use of antibiotics selects for resistance and creates a stable pool of vectors, enabling the transmission of resistant organisms among those admitted to the hospital and once they return home, the community [2]. Among resistant organisms, *Clostridioides difficile* is designated as an “urgent threat” by the CDC and is a major public health challenge both clinically and economically [1]. Vancomycin-resistant Enterococcus (VRE) is of slightly less concern (designated “serious threat”), but it is still a dangerous healthcare-associated pathogen because VRE strains are resistant to vancomycin, one of the antibiotics of last resort for many infections [1].

Antibiotics disrupt the colonization resistance provided by the healthy human gut microbiome [3]–[5]. There are many mechanisms by which the gut and its commensal bacteria provide colonization resistance. Many commensal bacteria produce small molecules or peptides, including bacteriocins, which target and kill other bacteria. Microcins are one such molecule. The probiotic, *Escherichia coli* Nissle 1917, produces microcins that inhibit the growth of pathogenic *E. coli* and *Salmonella enterica* in the inflamed intestine [6]. Commensal bacteria also interact with the host to induce the production of antimicrobial peptides or other molecules toxic to pathogens. A healthy commensal microbiome is required for the stimulation of the antimicrobial lectin, Reg3γ, which targets Gram positive pathogens in the intestine, including VRE [7]. Finally, a diverse microbial ecosystem modifies the environment in other ways that promote resistance to pathogen colonization. For example, *C. difficile* tends to be difficult to eradicate because it has the ability to form endospores, which are able to survive antibiotic treatment. One of the signals inducing germination of these spores is the presence of primary bile acids, but many commensal bacteria metabolize primary bile acids into secondary bile acids. Secondary bile acids can inhibit *C. difficile* spore germination, preventing infection [8]. These many mechanisms highlight the importance of the healthy gut microbiome in preventing infection.

Fecal microbiota transplants (FMTs) are one method that can restore the colonization resistance that is lost when the community is disrupted. Indeed, numerous published works have shown that FMT is able to restore the healthy and diverse microbial community of the gut and decrease the number of pathogens and antibiotic resistant bacteria in the intestine of both mice and humans [9]–[27]. FMT is a very promising therapy for decolonization and infection prevention, but it will be operationally challenging to use FMT as a prophylactic therapy on all patients who are at risk for infection. Currently, surveillance for presence of certain pathogens is performed in some institutions for some pathogens, but it is far from wide-spread [28]. It is logistically challenging to screen all patients for even a fraction of the pathogens they might be colonized with. A method that would allow us to identify patients that have microbiota disruptions, and are therefore at a higher risk for colonization and subsequent infection by any pathogen, could help identify patients that need to be treated with extra care, put into isolation, or treated with an FMT-like product that restores the healthy gut community, once one is approved by the FDA. To do this, we first need a better understanding of which microbiome predictors should be used to classify patients. This work is an early step toward that goal.

## 2. Materials and methods

### 2.1. Retrospective study

Previously banked samples from six academic centers where FMT was performed were used, including samples from a multi-center placebo-controlled trial (Table 1) [29]. Patients had multiply recurrent *C. difficile* infection and received either an allogenic FMT from a universal stool donor (OpenBiome, Somerville) or other healthy donor [29]. In the case of the placebo-controlled trial, each patient in the control group received an autologous FMT in which stool from the affected patient was infused back into the colon of the affected patient [29]. Stool samples were collected from patients prior to FMT and at one or more visits post-FMT. The primary endpoint was defined as recurrence of infection at 8 weeks. The exact time frame of the sample collections varied at different sites, but at least one sample was collected from almost all patients within 6-weeks of FMT.

### 2.2. Antibiotic resistance gene testing

Stool samples were sent to OpGen for antibiotic resistance testing, which was performed using OpGen's Acuitas MDRO gene test. This is a multiplex PCR test for common MDRO genes including VRE, CRE, and ESBL-E associated genes.

**Table 1.** Samples from six independent sites were screened for VRE colonization.

Principal investigator	Total patients	After exclusions for severe CDI	After exclusions for available timepoints	VRE + samples (no severe/complicated CDI)	VRE + samples receiving allogenic FMT	VRE + Samples receiving autologous FMT (control)
Brandt	18	18	16	5	1	4
Rodriguez	17	13	13	1	1	0
Kelly	21	21	21	6	3	3
Allegretti	17	16	16	4	4	0
Grinspan	7	6	6	2	2	0
Fischer	4	4	4	1	1	0
Total	84	78	76	19	12	7

### 2.3. Sample collection and 16S rRNA sequencing

Patients collected stool samples by sub-sampling approximately 1 gram of formed stool or 1 mL of liquid stool into 5 mL of RNALater. Samples were kept at room temperature for up to one week before being aliquoted and stored at −80 °C. Samples were thawed, RNALater was removed with PBS washing, and approximately 200 mg pelleted sample was aliquoted into 96 well Qiagen PowerBead Plates. DNA extraction, PCR amplification of the 16S rDNA V4 region, and Illumina paired end sequencing were performed at the University of Michigan core facility, as described previously [30].

### 2.4. 16S Processing

Primers were trimmed, paired ends merged, and operational taxonomic units (OTUs) identified with a custom in-house pipeline. OTUs represented in fewer than two unique samples and samples with fewer than 100 remaining reads were discarded. Taxonomic assignments for each OTU were called using UTAX trained on the Green genes 13_5 97% database. On average, there were 31,128 ± 13,316 reads per sample in the final OTU table.

### 2.5. Data analysis

Most data analysis was performed using in house python code, with the exception of the group significance test, which was performed using Qiime (http://qiime.org/scripts/group_significance.html) [31]. Alpha and beta diversity calculations were done using in-house code and the Scikit-Bio python package. Alpha diversity was calculated using the Shannon Index. Beta diversity was calculated using the Jensen-Shannon Divergence. The MDI for a sample was calculated by multiplying the average difference in alpha diversity (calculated using log2) between the sample and the healthy cohort by the average beta diversity between the sample and the healthy cohort. Based on the approximate range of MDI observed in healthy Finch stool donors, a healthy MDI score was defined as being less than 1. A dysbiotic MDI score is greater than 1, based on the calculation of the MDI for the patients with *C. difficile* infections (CDI) and using a publicly-available dataset consisting of patients undergoing chemotherapy and antibiotic treatment [32]. ROCs and AUCs were calculated and visualized using the Scikit-Learn python package.

## 3. Results

### 3.1. FMT prevents rCDI recurrence and decolonizes VRE

**Figure 1.**
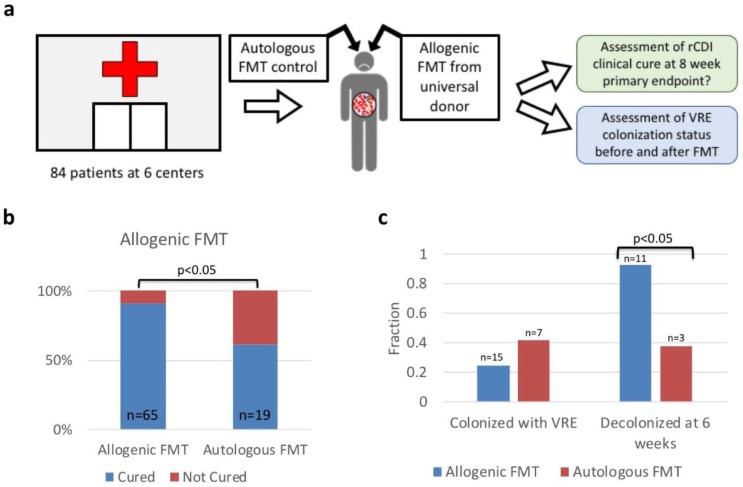
Data was collected from a multicenter retrospective study. (a) We obtained and performed 16S sequencing on a subset of samples collected from 84 patients at 6 centers. These patients all had rCDI. Some of these patients received an allogenic FMT from a universal donor at OpenBiome, while others received an autologous FMT as a control. Samples from before and after FMT were obtained for all patients. (b) Allogenic FMT was more effective at preventing recurrence of infection than autologous FMT. A total of 91% of patients who received allogenic FMT did not experience CDI recurrence. A total of 63% of patients who received autologous FMT did not experience CDI recurrence. This is within the usual placebo response range [33]. The difference between these groups is significant by Fisher's exact test. (c) Allogeneic FMT decolonizes VRE at 6 weeks. At baseline, ∼20–40% of rCDI patients were colonized with VRE. However, after 6 weeks, 92% of those initially colonized were decolonized in the group receiving allogenic FMT, while only 43% of those initially colonized in the group receiving autologous FMT was decolonized. The difference between these groups is significant by Fisher's exact test.

Our multi-center retrospective analysis (Figure 1a) included stool samples collected from 84 patients with rCDI that were enrolled at six independent sites (Table 1). Using samples from many different studies is advantageous because the use of multiple studies minimizes artefacts observed in the data due to geography or method of collection. A total of 65 patients received allogenic FMT from an OpenBiome universal donor and 19 patients received autologous FMT as a placebo treatment. We sequenced stool samples from before and up to two samples from after they received FMT using the 16S sequencing methods described above. Samples were tested for presence of VRE using the Opgen Acuitas® MDRO Gene Test, and colonization was defined as a positive result at one or more dilutions. The primary endpoint for clinical cure of rCDI was defined as prevention of infection recurrence at 8 weeks. The primary endpoint for VRE colonization was defined as clearance of VRE colonization at the first follow-up visit. The timing of the first follow-up visit varied but was less than 6 weeks after FMT for all patients.

FMT was an effective therapy for rCDI in our retrospective analysis (Figure 1b). Of the 65 patients with rCDI who received allogenic FMT, 59 (91%) achieved the primary endpoint of lack of recurrence at 8 weeks following FMT. In contrast, of the 19 patients in the control group who received autologous FMT, 12 (63%) were clinically cured at the primary endpoint (63%—This is within the usual range seen for placebo response in rCDI [33]). There was a statistically significant difference in recurrence between allogenic and autologous FMT groups (p < 0.05) by Fisher's exact test.

The majority of patients colonized with VRE were also decolonized after FMT. We found that 15 of 65 (23%) patients in the allogenic FMT group and 7 of 19 (37%) patients in the placebo group were colonized with VRE at baseline (pre-FMT). 3/15 (20%) of the VRE positive patients in the allogenic FMT group had severe or severe/complicated CDI and were excluded from subsequent analyses because of the significant physiological differences between standard and severe CDI, bringing the number of VRE positive patients receiving allogenic FMT to 12. At the primary endpoint (6 weeks post FMT) 11 of 12 (92%) colonized patients in the FMT group tested VRE negative compared to 3 of 7 (43%) in the control group (Figure 1c). The difference between allogenic and autologous FMT constitutes a statistically significant difference (p < 0.05 by Fisher's exact test).

### 3.2. Our simple microbiome disruption index can classify pre-FMT and post-FMT samples

We used this retrospective data to develop a microbiome disruption index (MDI). The goal of the MDI was to use broad descriptors of the microbiome community (alpha and beta diversity), so that we could use it to identify different types of dysbiosis in different populations. We used samples from the retrospective study (samples from patients receiving allogenic FMT where we had matched pre-FMT and post-FMT samples and the post FMT samples were from the timepoint less than 6 weeks post-FMT; samples from 38 patients in total) as well as samples from a healthy population, 63 donors from a universal stool bank (OpenBiome, Somerville, MA), to calculate the MDI. Stool donors are extensively screened for pathogens and risk factors similar to a blood bank, and other microbiome-mediated diseases [34].

**Figure 2.**
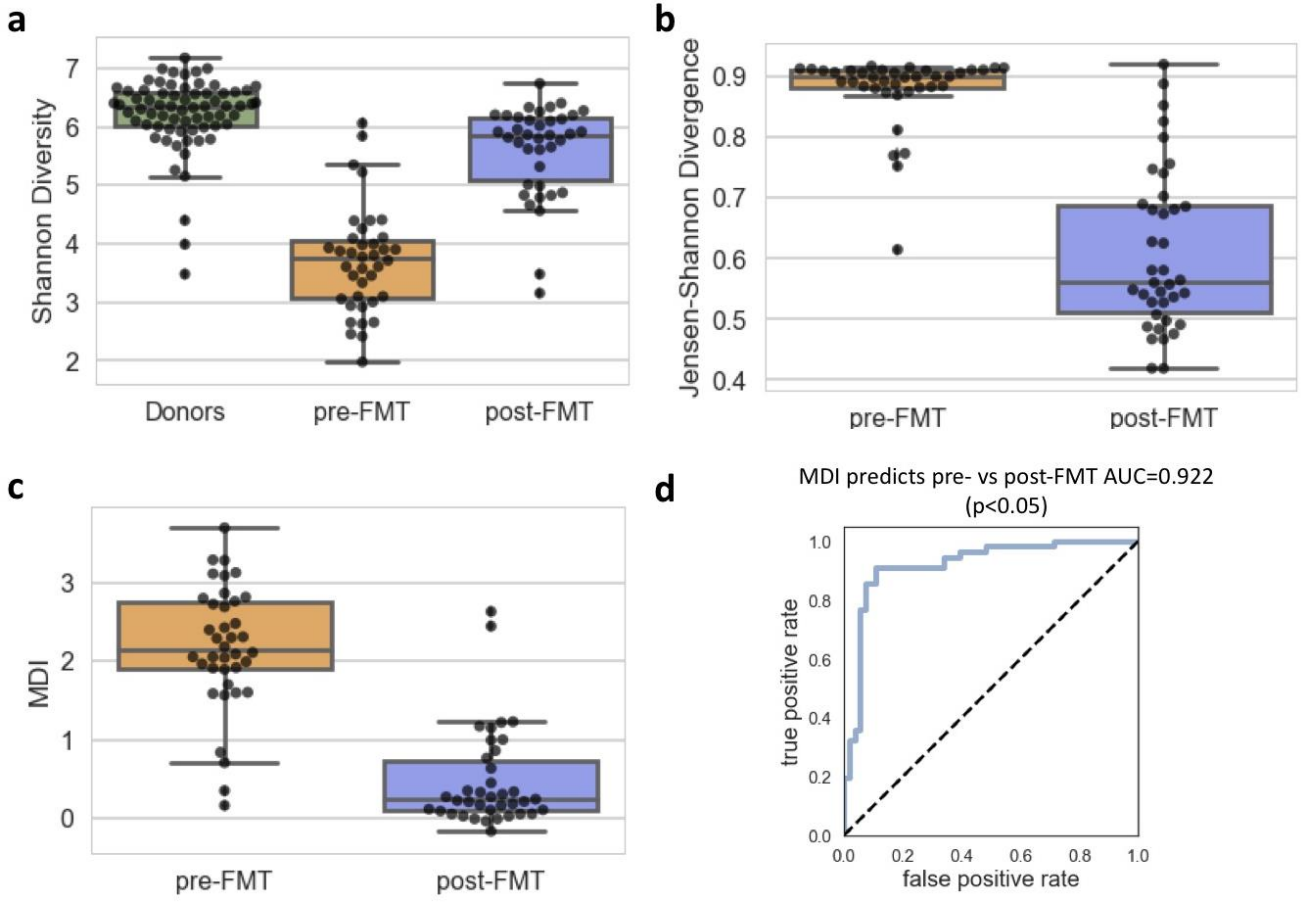
We developed a microbiome disruption index using data from patients receiving allogenic FMT. We used 16S sequencing data from patients receiving FMT where we had both pre-FMT and post-FMT sequencing data from a timepoint less than 6 weeks post-FMT, including both patients who were cured and those that continued to have recurrences. In total, this was matched pre and post FMT samples from 38 patient samples (the total number of patients was 65) and single samples from 63 healthy stool donors. (a) Alpha diversity, as measured by the Shannon Index was highest for the stool donors, similarly high for patient samples post-FMT, and lowest for patient samples pre-FMT. (b) Beta diversity, as measured by the Jensen-Shannon Divergence, compared to donors was highest for patient samples pre-FMT and was overall much lower for patient samples post-FMT. (c) The MDI was calculated by multiplying the average difference in alpha diversity between a sample and each of the stool donors by the average beta diversity between a sample and each of the stool donors. This calculation results in an MDI where undisrupted communities are generally found between an MDI of 0 and 1, and disrupted communities have an MDI of greater than 1. (d) ROC curves were used to determine whether the MDI could predict which patient samples were from pre-FMT vs post-FMT. The MDI classifies pre-FMT well, with an AUC of 0.922.

We used measures of alpha and beta diversity to calculate the MDI because these measures are known to be associated with microbiome disruption. First, we compared the alpha diversity of these populations because alpha diversity is often associated with microbiota community disruption, decreased in CDI patients, and known to increase after FMT [35],[36]. We chose to use Shannon's diversity index, a quantitative measure of total species richness, because it is commonly used with microbial datasets and robust to differences in sequencing depth. The Shannon index was calculated for each sample and donor, and the average difference in alpha diversity between the sample and each of the donors was used to describe the change from healthy levels of diversity. Because dysbiotic intestinal communities can be dominated by one or more high abundance species [32], we would expect that the diversity or species richness would be lower in patients pre-FMT than post-FMT and in healthy stool donors; our results support this statement (Figure 2a). Donors were much more diverse than pre-FMT patients, and post-FMT patients had a Shannon index that more closely resembled donors than patients pre-FMT.

Though we did observe a difference in the populations of patients pre-FMT and post-FMT using only alpha diversity, there have been cases described where patients with significant dysbioses have had high alpha diversity but a composition significantly different from that of a healthy person [35]. Therefore, we also included a measure of beta diversity in the MDI. The Jensen-Shannon divergence (JSD) is a method for assessing the distance between two probability distributions used to quantify differences between human microbiota communities. We used this method to look at the community divergence between patients with rCDI and healthy stool donors. Patients had samples taken before and after FMT, and the JSD was calculated for each sample and donor combination. The average JSD between the sample and each of the donors represents the average difference between the sample and a healthy community. We expected that post-FMT, the majority of patients would be cured of their CDI. Therefore, post-FMT, patients would more closely resemble the donors, and this was the case (Figure 2b).

We combined both alpha and beta diversity into the final MDI (Figure 2c). To combine these two measures, we calculated the average difference between alpha diversity of a sample and that of the healthy population. Then, we multiplied that value by the average beta diversity between the sample and the healthy population. The MDI calculated in this way is able to identify different types of community disruptions and is on a simple scale that generally ranges from 0–5. Using this and other published datasets [32], we have found that samples from healthy people usually have an MDI between 0 and 1, while those with a disrupted microbiome due to antibiotic treatment or infection have an MDI greater than 1.

Indeed, in this cohort, the majority of samples taken before FMT had an MDI greater than 1, and only 7 samples taken after FMT had an MDI greater than 1 (Figure 2d). Of these seven patients, 5 had decreases in MDI after FMT, and on average, these decreases were almost 1 (0.976). Because none of these 5 had any recurrent episodes of CDI, this suggests that FMT was effective at returning the healthy biodiversity of the gut, even if the MDI did not quite reach a healthy level. Of the other two patients, one had a pre-FMT MDI of less than 1 (0.705), and that fact combined with a relatively small increase in MDI post-FMT of 0.296 along with no CDI recurrences, suggests that this patient was already on the road to recovery. The final patient was the only one of these 7 who went on to have a recurrent CDI episode, and this patient also had the largest increase in MDI (0.851). These results suggest that it is not only MDI value of a sample that is important to consider but that the change in MDI over time may also help us better understand a patient's risk for disease.

We used a receiver operator curve (ROC) to describe the accuracy with which the MDI classified pre-FMT and post-FMT samples. The ROC plots the true positive rate of the model by the false positive rate of the model and calculates the area under the curve (AUC). A model that does no better than chance would have an AUC of 0.5, and a perfect model would have an AUC of 1. The MDI was able to predict pre-FMT vs post-FMT samples very accurately, with an AUC greater than 0.9 (Figure 2d).

### 3.3. MDI predicts which patients will develop a bloodstream infection

To confirm that the MDI calculation would be useful in other datasets, we used published data from Taur et al. 2012 [32] to calculate the MDI. In this dataset, the authors collected stool samples longitudinally from 94 patients undergoing hematopoietic stem cell transplantation (HSCT), while also tracking clinical data such as antibiotic use and development of bloodstream infections.

**Figure 3.**
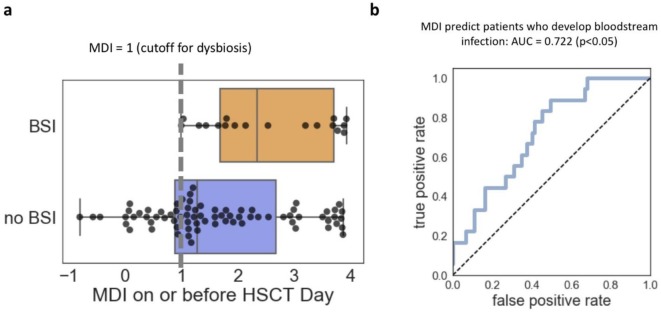
MDI predicts which patients develop bloodstream infection. (a) Patients who go on to develop bloodstream infection generally have an MDI greater than 1 on or before the day they received their stem cell transplant. (b) This fact allows us to predict which patients will develop a bloodstream infection, with a statistically significant AUC = 0.722.

If there were a way to identify which patients were at highest risk of developing bloodstream infection, those patients could be treated with extra care, put into isolation, or treated with an FMT to decolonize pathogens and decrease their risk of an infection. Therefore, for each patient, we identified the stool sample that was immediately before the stem cell transplant itself and calculated the MDI of that sample, using the same database of stool donors for the comparator as was used in the previous section. Then, we compared the MDI of the patients that did or did not go on to develop a bloodstream infection, and while the MDI of these two populations did overlap, the MDI for almost all those patients that went on to develop bloodstream infection was almost always greater than 1: the previously-defined maximum cutoff for a healthy MDI (Figure 3a). When confirmed with an ROC plot, we calculated an AUC of >0.7 (Figure 3b), suggesting that we can predict relatively accurately the patients that are at the highest risk of infection. This is especially exciting considering that in some cases, we were able to predict infection weeks before the infection actually developed. In summary, we were able to show that the MDI can be applied outside of FMT/*C. difficile* datasets, and the MDI could be used to identify the patients at highest risk of bloodstream infection. This result is remarkable. Because differences in sample preparation, sequencing, and patient population often create artefactual differences between datasets, cross-validation of a method such as this across different cohorts and datasets is generally very challenging.

### 3.4. The MDI does not predict VRE colonization state in patients with rCDI

**Figure 4.**
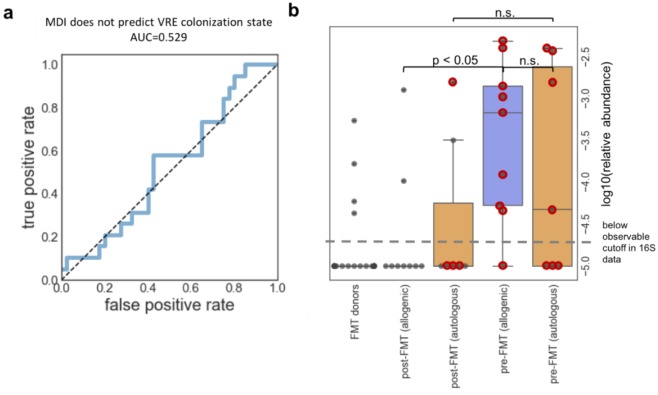
The MDI does not predict which samples are colonized with VRE. (a) Using only pre-FMT samples, the MDI was not able to predict which subjects were colonized with VRE. (b) This chart shows the relative abundance of Enterococcus across VRE-colonized samples pre-FMT and samples from the same patient post-FMT. VRE-colonized samples are circled in red. While there was no difference in Enterococcus abundance between pre-FMT samples from patients receiving autologous and allogenic FMT (using the Mann-Whitney U test), after FMT we observed a significant decrease in the average relative abundance of Enterococcus for only patients receiving allogenic FMT. There was not a statistically significant decrease in the post-autologous FMT samples (as measured using the Mann-Whitney U test). Notes: None of the FMT donors were VRE positive, but Enterococcus abundance in donors is shown for reference. In addition, we do not have 16S data from all the allogenic FMT samples, which is why the single VRE-colonized sample post-allogenic FMT is not shown.

Vancomycin is a standard of care treatment for rCDI, and VRE is the most notorious vancomycin-resistant pathogen. Because patients with rCDI are already experiencing severe disruption of the gut microbiota [37],[38], and are regularly treated with many courses of oral non-absorbable vancomycin, rCDI patient colons can become environments that select and enrich for vancomycin-resistant pathogens like VRE. Therefore, we investigated whether patients who were co-colonized with *C. difficile* and VRE experienced additional disruption of the microbiome. For this analysis, we only compared patient samples from before they received either intervention (allogenic or autologous FMT), and based on the MDI of samples pre-FMT, we could not predict whether patients were colonized with VRE (Figure 4a). Though there is little difference in the extent of the dysbiosis as measured by the MDI between subjects with rCDI and those with rCDI and colonized with VRE, this does not mean that in other populations, there is no difference in community disruption between those colonized with VRE and those not colonized. In this light, it would be more informative to compare those colonized and not colonized post-allogenic FMT when CDI is cured, but only one subject was VRE colonized after allogenic FMT in this dataset, so a meaningful comparison cannot be made. With a larger dataset, we will be able to more accurately assess the role of VRE in microbiome disruption.

Previous work in different patient populations has shown that antibiotic treatment and subsequent VRE colonization can result in Enterococcal domination of the gut and a significant decrease in diversity [32],[39], and we asked whether this was also the case in this population. So, we examined the data to assess the relative abundance of the Enterococcus genus across the different VRE positive samples. We measured the total relative abundance of all Enterococcus strains in the gut of the VRE positive samples before and after intervention. It is impossible to identify VRE using 16S sequencing because the vancomycin resistance gene, *vanA*, is not sequenced and because it is difficult to distinguish Enterococcus species using only the 16S gene (for example *E. casseliflavus* and *E. gallinarum* have 99.9% identical 16S sequences [40],[41]), so we used Enterococcus abundance as a proxy. We can do this because the vast majority of healthy donors have Enterococcus at relative abundance levels below the threshold that can be observed from 16S data. In contrast to the previous work described above, we found that Enterococcus abundance was very low in both VRE colonized and decolonized patients, with relative abundances never greater than 1%. However, Enterococcus abundance was increased in VRE colonized samples compared to healthy donors (Figure 4b). Furthermore, though there was little difference between the abundance of Enterococcus before FMT, the average relative abundance of Enterococcus in the post autologous FMT groups is higher than in the post allogenic FMT group (Figure 4b).

### 3.5. Relative abundance of Enterobacteriaceae predicts VRE colonization

Because the MDI does not predict VRE decolonization, we asked whether there are specific bacterial taxa whose presence or relative abundance could better predict decolonization. We compared VRE positive and VRE negative samples pre-intervention and post-intervention separately to control for the community differences associated with FMT. We used QIIME's group significance tool to identify taxa that were significantly different between groups, and we identified a number of taxa that were significantly enriched in the VRE colonized samples, though none were significantly enriched after multiple hypothesis correction (Table 2). We noticed that many of these taxa belonged to the phylum Proteobacteria, and we found this particularly interesting because the relative abundance of Proteobacteria in healthy stool samples is known to be generally low [42], but the relative abundance of Proteobacteria in VRE-positive samples to exceed 80% in some cases.

**Table 2.** Taxa found to be more abundant in VRE colonized samples pre and post FMT.

Family	Genus	Pre or Post FMT	Fold increased abundance in VRE-colonized samples	p-value
Lachnospiraceae	Lachnospira	Pre	454	0.001
Rikenellaceae	N/A	Pre	9.48	0.003
Bacteroidaceae	Bacteroides	Pre	168	0.007
Enterobacteriaceae	N/A	Pre	3.78	0.021
Enterobacteriaceae	Escherichia	Pre	8.53	0.025
Neisseriaceae	Neisseria	Pre	110	0.038
Porphyromonadaceae	Parabacteroides	Pre	104	0.041
Tissierellaceae	Finegoldia	Post	27.8	0.008
Unnamed Burkholderiales	N/A	Post	13.3	0.004
Enterobacteriaceae	N/A	Post	7.26	0.016

**Figure 5.**
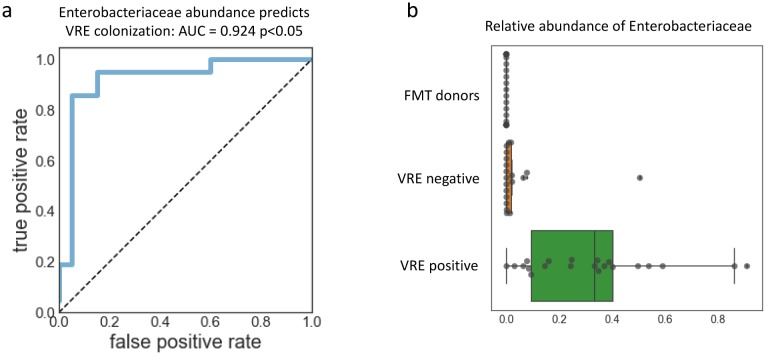
Enterobacteriaceae abundance predicts VRE colonization in the pre-FMT samples. The group significance test suggested that Proteobacterial strains may predict VRE colonization. (a) We tested every Proteobacterial family to identify those that could predict VRE colonization. Of all the families, only one, Enterobacteriaceae, had an AUC greater than 0.7. (b) Most of the VRE positive samples had a very high relative abundance of Enterobacteriaceae; in some cases making up greater than 80% of the total community, while in VRE negative patients and in the FMT donors, the relative abundance of Enterobacteriaceae in the samples is very low.

Diagnostics measuring the abundance of specific taxa associated with a disorder or disease could be used in the future to predict whether a patient has that disease or is likely to develop that disease. Therefore, we asked which Proteobacterial family was best able to predict which patients were colonized with VRE pre-FMT. We again used a ROC to assess how well each family predicted VRE colonization state. We found that only one family, Enterobacteriaceae, had an AUC greater than 0.7, and it was able to predict VRE colonization very accurately, with an AUC of 0.924 (Figure 5a). In fact, the high abundance of Proteobacteria seems to be driven almost entirely by Enterobacteriaceae. The majority of the VRE colonized patients have guts dominated by Enterobacteriaceae (relative abundance greater than 30%), with some exceeding 80% (Figure 5b). Perhaps, in the future, patients with rCDI could be screened for increased Enterobacteriaceae abundance in their stool, to identify whether they were at risk for VRE or other pathogen colonization.

## 4. Discussion

### 4.1. The MDI could identify subjects with rCDI but not those colonized with VRE

In this manuscript, we have described two different methods that are able to classify sample and patient types. We used alpha and beta diversity measures to develop a MDI, and we found that broad community measures such as those that were used to calculate the MDI were sufficient for predicting pre- vs post-FMT status and risk of bloodstream infection in HSCT patients but not VRE colonization. This highlights the magnitude of the community disruptions that occur as a part of, and often prior to, *C. difficile* infection and HSCT transplantation, as well as the positive effect that FMT has on returning the disrupted community to its healthy state. This can be seen in our analysis, as the MDI we developed can distinguish between pre-FMT and post-FMT samples.

However, we found that the MDI was not readily able to identify which patients were colonized with VRE in the background of rCDI. Instead, we found that abundance of Enterobacteriaceae was predictive of VRE colonization status. This suggests that although we hoped that the MDI would be a global indicator of dysbiosis, it may be most effective for severe dysbioses, such as those associated with enteric infections like rCDI or severe disturbances from the chemotherapy and antibiotic treatment that precede HSCT. In the future, this MDI and other microbiome measures may be a useful biomarker for assessing risk of developing a disease and prioritizing those patients for different treatments.

### 4.2. Enterobacteriaceae blooms are associated with pathogen colonization

Blooms of facultative anaerobes, particularly Enterobacteriaceae, are associated with inflammatory conditions in the intestine, such as those caused by CDI or inflammatory bowel disease [43],[44]. The healthy colon is almost completely anaerobic, and there, obligate anaerobes rely on fermentation of carbohydrates and amino acids to generate energy. Byproducts of this process include the short chain fatty acids, which are thought to have important roles in maintaining intestinal epithelial integrity and supporting an anti-inflammatory state [45]–[47]. However, during inflammation, the host generates more electron acceptors, including reactive oxygen species (ROS) and reactive nitrogen species (RNS). The makes the gut, particularly close to the epithelium, much more aerobic, inhibiting the growth of the obligate anaerobic community, and leaving a niche available for the facultative anaerobic bacteria in the community [48].

There is evidence that inflammation is associated with an increase in Enterobacteriaceae abundance. Enterobacteriaceae are not only able to grow in aerobic conditions, but they are much more likely to be able to utilize RNS produced by the host for energy production, through nitrate respiration [49]. In addition, presence of host nitrate also allows Enterobacteriaceae to more easily utilize metabolic endproducts of other commensal strains for energy production [50]. Finally, some members of Enterobacteriaceae can use other molecules produced as byproducts of host inflammation. For example, in the presence of tetrathionate (another host-derived electron acceptor), *Salmonella enterica* can use ethanolamine (a byproduct of phospholipids released from inflamed host cells) as a sole carbon source [51].

Therefore, it is not surprising that we observe increased Enterobacteriaceae in this population. CDI causes significant inflammation and injury to the colonic epithelium, releasing many of the nutrients described above. As another facultative anaerobe, perhaps VRE also prefers more inflamed environments and contributes to the inflammation itself, increasing the available niches for Enterobacteriaceae strains. *Enterococcus/*VRE and *C. difficile* have previously been found to associate in the context of rCDI [52]–[54]. Perhaps co-colonization with VRE and *C. difficile* results in synergistically increased inflammation. Another hypothesis is that perhaps VRE and Enterobacteriaceae strains form a mutually beneficial metabolic relationship, allowing both strains to flourish in the inflamed colon. Future research will shed light on this observation.

## 5. Conclusions

In conclusion, we were able to use data from a multicenter retrospective study to develop a MDI that reliably classifies patients with pre-FMT rCDI. Extraordinarily, this simple index was also able to predict which HSCT patients would develop blood stream infections, which validates this method in a completely different cohort, and shows that it can be used broadly to identify dysbiosis in a number of different indications. While other approaches have previously developed strong classifiers based on complicated multi-variate models [55], to our knowledge, this is the first index that can classify microbial disruptions across indications. We were also able to identify specific clades that appear to be associated with VRE colonization, a subtler community change. This work is important for understanding microbial dynamics associated with colonization by different pathogens, and further work will be focused on better understanding the mechanisms underlying these observations and on developing a more complex MDI that might be able to capture a wider variety of disrupted ecologies. With a better understanding of the taxa associated with pathogen colonization and decolonization, these or similar methods could be used to help identify patients who are at risk for colonization and infection, and microbial therapeutics, such as FMT, could be used to restore the healthy microbiome and prevent life-threatening infections.
